# Identifying important ecosystem service areas based on distributions of ecosystem services in the Beijing–Tianjin–Hebei region, China

**DOI:** 10.7717/peerj.13881

**Published:** 2022-08-18

**Authors:** Cuiyun Cheng, Shuping Zhang, Meichun Zhou, Yanchun Du, Chazhong Ge

**Affiliations:** 1 Chinese Academy of Environmental Planning, Beijing, China; 2 Zhejiang Zhongshui Engineering Technolgy Co., Ltd, Hangzhou, China; 3 Changzhou Environmental Protection Research Institute, Changzhou, China

**Keywords:** Water conservation, Soil conservation, Sandstorm prevention, Biodiversity importance, Beijing–Tianjin–Hebei Region

## Abstract

Water conservation, soil conservation, biodiversity importance, and sandstorm prevention are important ecosystem services (ES) and the core challenges to sustainable economic and societal development in the Beijing–Tianjin–Hebei (BTH) region. Using the Integrated Valuation of Ecosystem Services and Tradeoffs (InVEST) model and observation data, we identified high-value ES areas in the BTH region. The high-value ES areas were mainly found in the northern and southwestern parts of the region, like the Yanshan Mountain Range and the Taihang Mountain Range. The ecosystem in the northern mountains is dominated by forest and grassland, and generally provides more valuable ES than does the eastern agricultural plain. Greater species richness was mainly found in the northern mountains with low human activity intensity. Due to its proximity, the Yanshan Mountain Range is critical to the health of the local ecosystem of Beijing. High biodiversity was present in the vicinity of the national nature reserves. Compared with other regions of China, changes in the BTH region are highly intense. Reinforcement of biodiversity conservation and ecosystem restoration in areas with a high degree of ES in the BTH region are capable of effectively improving habitat quality and regional ES.

## Introduction

Ecosystem services (ES) refer to the products and services provided by ecosystem structures and processes for human society so that humans may obtain the benefits from an ecosystem, directly or indirectly (Daily, 1997; Costanza et al., 1997; Costanza et al., 2011). ES are considered as a bridge between natural and social ecosystems. Spatial assessments of ES can be used in ecosystem conservation to provide the long-term ES supply (Bryant et al., 2018). ES hotspots are defined as regions with high service diversity, high service biophysical or monetary value, or high service supply capacity (Schröter & Remme, 2016; Li et al., 2017; Daryanto et al., 2018). Here, we focus on hotspots and define them as areas of high biophysical value for a single service. Identifying hotspots can offer a reference for scientifically defining conservation boundaries and setting conservation priority area when allocating limited resources in the process of ecosystem management (Cao et al., 2007).

ES are crucial for human wellbeing (Felipe-Lucia et al., 2020). Evaluation of ES is also the theoretical basis for ecological restoration and benefit evaluations, biodiversity protection, green development, ecological compensation mechanism, and response to global climate change, and thus a major strategic requirement to ensure national ecological security (Asadolahi et al., 2018). There is a growing awareness that human activities are constantly changing the structure and function of ecosystems and weakening ES (Bai et al., 2018). However, there is very little ecological information to provide a basis for knowledgeable decision making in ecosystem conservation and management. Thus, scientists and managers are struggling with key components of ecosystem management, determination of boundaries and scope of management, and relevance of management methods (Groffman et al., 2006; De Groot et al., 2010). Such unknowns directly impact ES conservation and management (Feng et al., 2015).

The biodiversity of organisms and their environment is complex, involving the synthesis of various related ecological processes and an understanding of the relations of organisms to one another and to their physical surroundings (Brevik et al., 2018). Biodiversity has always been the main goal of natural resources conservation, implementation, and management (Yang et al., 2018a; Yang et al., 2018b). Currently, there is a shift in ecological conservation from a focus on biodiversity to the provision of ES (Watson et al., 2019). Well-designed protected areas allow the coexistence of human activities and nature for improvements in the wellbeing of both (Zhang et al., 2018). Evidence indicates that the division of key regions into their specific ES not only guarantees biodiversity, but also provides adequate ES and enhances ecosystem resilience (Dai et al., 2018). In China, there is a critical need to address conservation requirements for biodiversity preservation and ES (Kang et al., 2017).

The relationship between ES provided by the natural ecosystem and the ecological response to human activities may extend beyond the carrying capacity of the ecosystem and include natural disasters, heat island effects, and pollution (Wang et al., 2011; Xu et al., 2017). Conversely, there is a relationship between environmental pressure and ecological damage caused by human activities and active ecological construction (Gao et al., 2011). Ecological restoration requires considerable capital investment although it does not necessarily achieve satisfactory ecological benefits (Steinman et al., 2017). It is necessary to understand the relationship between biodiversity conservation priority areas and ES, and to reconsider how to implement ecological restoration with respect to biodiversity and ES (Kong et al., 2018). Identification of multiservice areas of ES can provide a scientific basis and data support to improve the efficacy of such projects. In this study, we focus on the Beijing–Tianjin–Hebei (BTH) region to determine important ES areas that can serve to advance regional ecological restoration planning.

Ecological restoration is a process to help a degraded ecosystem recover, with the goal to return to a state similar to the original state. In China, ecological restoration has become particularly important as a result of large-scale land degradation due to climate change and unsustainable human use. Artificial recovery emphasizes the positive role of humans in the recovery process, but natural processes such as succession that affect recovery success over time also must be taken into account. Recovery also can be primarily natural: natural processes may be sufficient to restore the ecosystem to its original state if an ecosystem is protected from further degradation (Carpenter et al., 2009).

Beijing is the capital of China and is rich in many resources, however, the BTH region currently faces a double threat from water and mineral resource shortages (Baskaran, Cullen & Colombo, 2010). Few studies have quantitatively estimated biodiversity and ES with respect to priority area selection and planning management. A central approach to countering the threats is protected areas (PA) establishment, such as nature reserves or national parks, to limit human influences and protect the geographical space of nature. Biodiversity has traditionally been a major objective of the design, implementation, and management of natural area through the International Union for Conservation of Nature (IUCN). The IUCN definition of protected areas includes an explicit reference to the protection of “nature with associated ecosystem services”. Biodiversity has historically been a primary goal of protected area design, implementation, and management. A major shift is now underway, expanding the objectives of protected areas from a primary focus on biodiversity to also include the provision of ES for human wellbeing. Well-designed protected areas can harmonize people and nature and improve the wellbeing of both. Achieving simultaneous biodiversity and ES preservation is a top priority. Most nature reserves in China do not have a clear planning framework to maximize the efficiency and representation of conservation objectives. This is a significant gap, and the spatial overlap between biodiversity goals and ES is low (Zhang et al., 2010). How to form a new ecosystem resource management model to promote ES and reduce the impact of humans on the ecological environment is an important scientific issue that urgently needs research today (Miao et al., 2019).

The weakness of the study on ecological vulnerability will be addressed through analysis of the important areas of biodiversity and ES in the BHT Nature Reserve. We overlay the threatened biodiversity map with other maps that primarily regulate ES. We have chosen these four ES (water conservation, soil conservation, biodiversity importance, and sandstorm prevention) because they are the national priorities of policymakers and the foundation of human wellbeing. Then, we propose the establishment of the BHT protected area to address the deficiencies of the existing protection in the BHT region. It is hoped that this method can alleviate the serious ecological problems in the BTH area to a certain extent.

## Methods

### Study site

**Figure 1:**
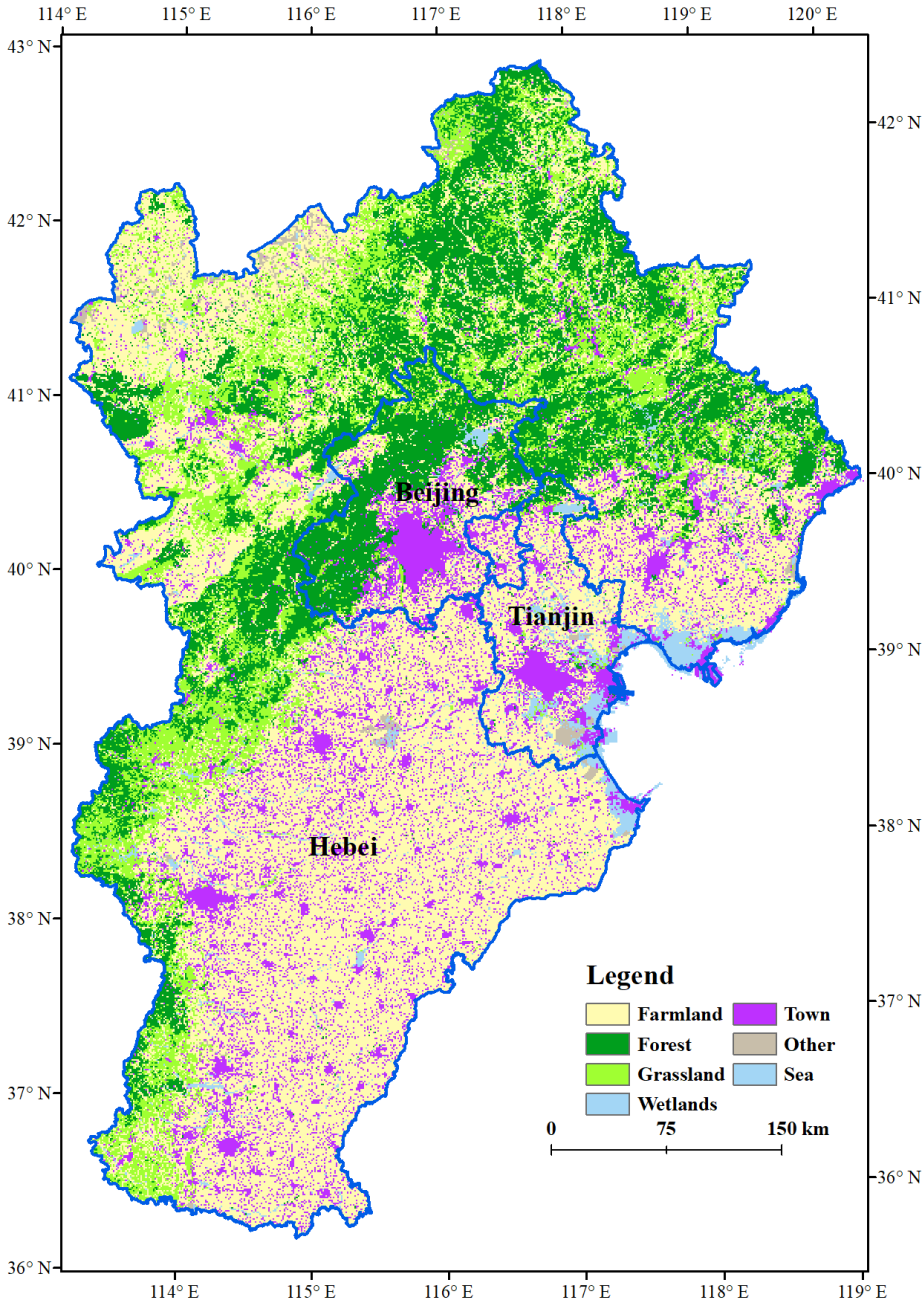
Map of Beijing-Tianjin-Hebei region and distribution of land use in year 2015.

The BTH region includes two province-level municipalities (Beijing in the center and Tianjin in the east) and one province (Hebei) (Zhu et al., 2018) (Fig. 1). The vast hinterland of the economically vibrant BTH region has significant development potential (Zhao et al., 2011).

**Figure 2:**
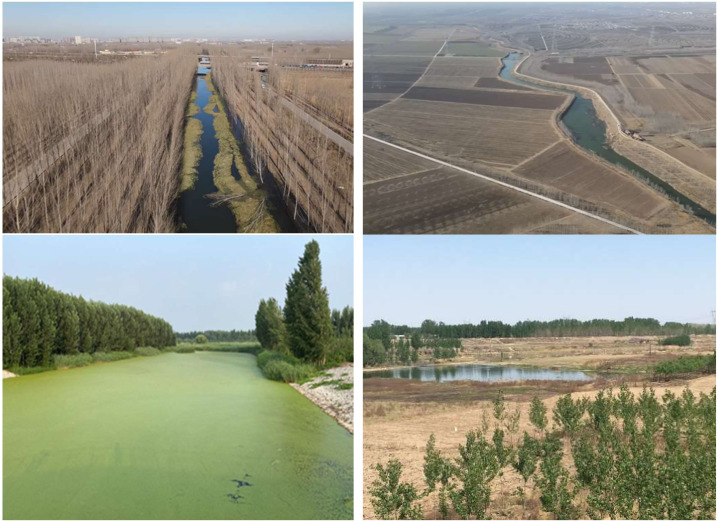
Examples of typical environments in the study area. (A) The woods near the river in Tianjin. (B) The plain area of Hebei. (C) The typical river course of Beijing. (D) The cash crop forest in the suburbs of Hebei.

The terrain is tilted from northwest to southeast and is high in the north and west and low in the south and east (Zhang et al., 2019). The Taihang and Yanshan mountains are important ecological barriers to the BTH region, which protect the integrity of ES. The southeastern plains are the main grain district of the BTH region (Fig. 2).

The increasing intensity of human activities exerts pressure on an ecosystem. Environmental degradation has not been effectively curbed. The local government has not taken effective measures to slow the trend of environmental declines. In cities other than Beijing and Tianjin, urban pollution is serious, and soil erosion in the Taihang and Yanshan mountains is increasing. Further, the plains are shrinking and disappearing, coastal and estuarine ecosystems are degrading, the land is subsiding, and seawater is intruding, causing increasing threats to habitat quality and sustainable ecological development (Yang et al., 2018a; Yang et al., 2018b).

### Data sources

Land coverage data of the BTH region in 2000 and 2010 were obtained from a global land coverage product with 30-m resolution (GlobeLand30) (Xie, He & Xie, 2017). The images used for classifying land into cover types included Landsat TM 5 and ETM+ multispectral imagery from the China Environmental Disaster Reduction Satellite (HJ-1). Land cover is divided into seven types: forest, shrub, grassland, wetland, farmland, urban construction land, and bare land. The Shuttle Radar Topography Mission (SRTM) 90-m spatial resolution digital elevation model (DEM) was used to determine water bodies and to divide the watersheds (Norse & Ju, 2015). Data were converted based on the pixel binary model. Vegetation coverage was calculated on the basis of the Moderate Resolution Imaging Spectroradiometer (MODIS) data product (Adole, Dash & Atkinson, 2016). The vegetation index–biomass and Net Primary Productivity (NPP) were used to estimate vegetation biomass (Bentsen, Lindholst & Konijnendijk, 2010). The vegetation index–biomass is converted from remote sensing data; the cumulative NPP method uses the growth period of grassland or farmland (starting and ending growth times) to calculate the accumulated aboveground biomass (Cleal et al., 2012). Meteorological data include the average precipitation, temperature, and total solar radiation from 2000 to 2010. These data were collected from individual meteorological stations (Table 1).

**Table 1:** Sources of principal data.

Data name	Data resolution	Data source
Land cover map	90 m	Chinese Academy of Sciences Institute of remote sensing
Soil map	1:1,000,000	Chinese Academy of Sciences Institute of remote sensing
SRTM digital elevation model	90 m	International scientific data service platform
Precipitation, temperature and total solar radiation	0.05°	Chinese National Metrological Information Center/China Meteorological Administration (NMIC/CMA)
MODIS-NDVI	250 m	Land Processes Distributed Active Archive Center (LP DAAC)
Validation data	Counties, watersheds, quadrats, points	Water resources bulletin, soil & water conservation bulletin, hydrometric station, published works, Chongqing Government

### Research methods

#### Remote sensing–based assessment of ecosystem service

The data for this work came from national ecosystem assessment projects and national key infrastructure projects. Water yield was calculated and defined as water conservation capacity (Cao et al., 2017). The water conservation capacity of a reservoir is estimated using the water balance equation of the model, which is the difference of the sum of precipitation, runoff, and evapotranspiration. The sandstorm prevention ES was obtained from the revised wind erosion equation (Czemiel Berndtsson, 2010). Based on the Integrated Valuation of ES and Tradeoffs (InVEST) model, we estimated the extent of habitat, vegetation types, and degradation status of the landscape.

#### Soil conservation

Generally speaking, soil conservation is the difference between actual soil erosion and potential soil erosion (De Sy et al., 2012). Several key factors of soil erosion are rainfall erosion rate, soil erodibility, topography, vegetation, and protection measures:

\begin{eqnarray*}SC=R\times K\times LS\times \left( 1-C\times P \right) . \end{eqnarray*}

In this formula, SC is soil conservation capacity; R is annual rainfall erosion capacity; K is soil erodibility; LS is a dimensionless terrain, which reflects the influence of slope length and steepness on soil erosion; C is a dimensionless vegetation factor; and *P* is the dimensionless conservation practice (Dvorak & Volder, 2010).

#### Water conservation

The water yield function (a proxy for water conservation) was calculated using the InVEST model as follows (Flindt et al., 2016):

\begin{eqnarray*}WY= \left( 1- \frac{1+Z\times \frac{AWC}{{P}_{a}} \times \frac{kE{T}_{0}}{{P}_{a}} }{1+Z\times \frac{AWC}{{P}_{a}} \times \frac{kE{T}_{0}}{{P}_{a}} + \frac{P}{kE{T}_{0}} } \right) \times {P}_{a} \end{eqnarray*}

where, WY is annual water output per pixel, P_a_ is annual precipitation per pixel, AWC is the effective soil water content of plants, *Z* is a seasonal factor representing seasonal rainfall distribution and rainfall depth, k is the vegetation evapotranspiration related to land coverage type, and ET_0_ is the reference evapotranspiration calculated by the Hargreaves formula. The *Z* value of 3.0 indicates the similarity of the amount of water yield to that of natural runoff (Gabel et al., 2016).

#### Sandstorm prevention

Sandstorm prevention (wind erosion control service) refers to the sand retained in an ecosystem within a certain period. Sandstorm prevention capacity was calculated as sand loss from areas without vegetation cover (potential sand loss, S _LP_) minus that in current land use/land cover type (actual sand loss, S_L_). We used the revised wind erosion equation (RWEQ) model to estimate the sandstorm prevention service. The formulas are as follows:

\begin{eqnarray*}G& ={S}_{LP}-{S}_{L} \end{eqnarray*}

\begin{eqnarray*}{S}_{LP}& = \frac{2z}{{S}_{P}^{2}} \times {Q}_{\max P}\times {e}^{-(z/{s}_{P})^{2}} \end{eqnarray*}

\begin{eqnarray*}{S}_{P}& =150.71\times (WF\times EF\times SCF\times {K}^{{}^{{^{\prime}}}})-0.3711 \end{eqnarray*}

\begin{eqnarray*}{Q}_{\max P}& =109.8\times [WF\times EF\times SCF\times {K}^{{}^{{^{\prime}}}}] \end{eqnarray*}

\begin{eqnarray*}{S}_{L}& = \frac{2z}{{S}^{2}} \times {Q}_{\max }\times {e}^{-(z/s)^{2}} \end{eqnarray*}

\begin{eqnarray*}S& =150.71\times (WF\times EF\times SCF\times {K}^{{}^{{^{\prime}}}}\times C)-0.3711 \end{eqnarray*}

\begin{eqnarray*}{Q}_{\max }& =109.8\times [WF\times EF\times SCF\times {K}^{{}^{{^{\prime}}}}\times C] \end{eqnarray*}

In these formulas, G (kg m^−2^) is sand control capacity, S_L_ (kg m^−2^) is soil erosion caused by wind, S_LP_ (kg m^−2^) is the potential soil erosion caused by wind, WF (kg m^−1^) is a climate factor, K’ is the surface roughness factor, and S (m) is the critical field length (Habeck-Fardy & Nanson, 2014).

#### Biodiversity importance

The biodiversity model has inherent spatial features, and biodiversity can be evaluated by analyzing the threat to biodiversity in the land coverage map (Haynes, 2009). For example, in a fire-prone ecosystem, one would define the sensitivity to fire. The land use change j Field x habitat quality (Q_xj_), is calculated as follows (Qureshi et al., 2012):

\begin{eqnarray*}{Q}_{xj}={H}_{j}\times \left( 1- \left( \frac{{D}_{xj}^{z}}{{D}_{xj}^{z}+{k}^{z}} \right) \right) \end{eqnarray*}

where H_j_ is the chosen conservation objective based on land use/land cover (LULC) user type, which mainly provides habitat for the selected protection targets in the study. In the parameter ${D}_{xj}^{z}$, j is the total threat level in a grid cell x; z and k are scaling parameters. The detailed calculation formula for *D*_*xj*_ is described in the InVEST user guide.

#### Statistical analysis

In order to examine the relationship between simulation results and observational data, Pearson correlation analysis was used to analyze county, quadrat and watershed as statistical units respectively. We used the statistical analysis in SPSS 17.0.

### Ecosystem service mapping

The importance of ES in a given region depends on the importance of individual ES within the region. Considering the irreplaceable role of each ES function in ensuring regional ecological security and supporting social and economic development, it is considered that only one ES function is very important in a given region, so this area is an important area of that ES function. The maximum value of the importance of each individual ES function is assigned to the comprehensive evaluation of the importance of ES functions, based on this principle.

Grid maps of different types of ES were obtained based on the model calculations. We used the Raster Calculator in ArcGIS to obtain a raster map of the normalized ES function values. We exported the raster data attribute table, which records the ES value of each raster pixel; arranged the service value in descending order; and calculated the cumulative service value. The raster values corresponding to 50% and 80% of the total ES value are used as the demarcation points of ES evaluation and classification. The reclassification tool of ArcGIS software is used to divide the importance of of each ES into four levels, as the top 50% (extremely important), 50–75% (important), 75–90% (moderately important), and 90–100% (general). The spatial distribution pattern of the importance of ES functions in the BHT region was obtained.

Using soil conservation as an example, the grading steps were as follows: first, calculate the soil holding capacity of each sorted grid; then sort the grids in descending order of soil holding capacity, and calculate the cumulative ratio of soil holding across the grid unit. The importance of each service function was superimposed and integrated by importance into comprehensive ES indexes (Fu & Wei, 2018).

### Gi* statistics-based hotspots analysis

In the study of ES hotspots, hotspot areas are those with high service aggregation, so spatial correlation analysis can be used to identify hotspots and establish protected areas for these hotspots, which can effectively protect biodiversity and ES. The Gi* statistic represents the amount of spatial clustering within a local sample and can be calculated as the sum of the differences between the local sample value and the mean. The Gi* coefficient is a commonly used local spatial autocorrelation index based on the full distance matrix. It can detect which areas in the study area are highly clustered, that is, hotspot areas (Fu & Wei, 2018). The standardized Gi* statistic for each feature in the data set represents a *z*-score. *Z*-scores are measures of standard deviation, and *p* values are probabilities.

In this work, the spatial autocorrelation analysis of ArcGIS software was used to calculate the aggregation degree of biodiversity changes. As a tool integrated in ArcGIS 10.2, this method takes each raster pixel in the context of adjacent elements into the calculation and outputs a new feature class with *z*-score, *p*-value, and confidence. Features with high *z*-scores and small *p*-values represent statistically significant hotspots. The magnitude of the absolute value of the *z*-score explains the strength of the clustering. This approach can help identify hotspots with different levels of prominence, so stakeholders can prioritize accordingly based on actual needs.

To simplify the analysis, the BTH region was divided into a number of small areas with polygon sizes of 10,000 hectares as hotspot analysis statistical units. The degree of aggregation of biodiversity change was calculated with spatial autocorrelation analysis in ArcGIS software (Xiao & Xiao, 2019). The computational formula for Getis–Ord G_i_* (*z*-score) is as follows:

\begin{eqnarray*}{G}_{i}^{\ast }& = \frac{\sum _{j=1}^{n}{w}_{i,j}{x}_{j}-\overline{X}\sum _{j=1}^{n}{w}_{i,j}}{S\sqrt{ \frac{ \left\vert \sum _{j=1}^{n}{w}_{i,j}^{2}-(\sum _{j=1}^{n}{w}_{i,j})^{2} \right\vert }{n-1} }} \end{eqnarray*}

\begin{eqnarray*}\overline{X}& = \frac{\sum _{j=1}^{n}{x}_{j}}{n} \end{eqnarray*}

\begin{eqnarray*}\mathrm{S}& =\sqrt{ \frac{\sum _{j=1}^{n}{x}_{j}^{2}}{n} -{ \left( \overline{X} \right) }^{2}} \end{eqnarray*}

where the *G_i_ is a *z*-score of patch i. x_j_ is the attribute value for patch j; w_ij_ is the spatial weight between patch i and patch j, if the distance from a neighbor j to the feature i is within the distance, w_ij_ = 1; otherwise w_ij_ = 0; n is the total number of grid cells and identifying and mapping the hotspots can visualize priority areas spatial-explicitly, which is helpful for targeted policy making.

## Results

### Model validation

**Figure 3:**
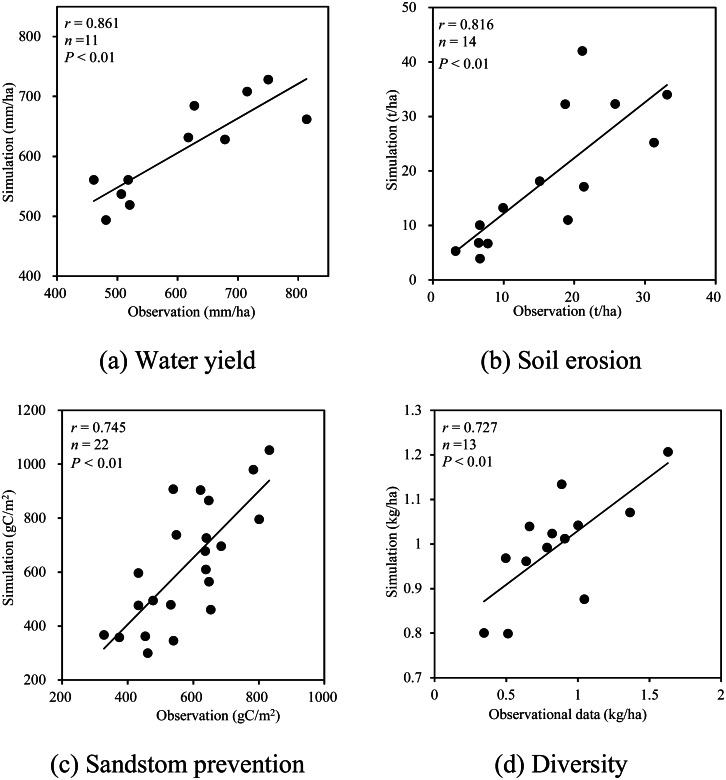
Correlation validation of the ecosystem service model. (A) Water yield. (B) Soil erosion. (C) Sandstom prevention. (D) Diversity.

We used the Pearson correlation coefficient and observation-based data to validate the model. The results showed that the modeled values were highly correlated with measurement data, indicating that the ES model in this research simulated those in the study (Fig. 3).

### Types of land use and potential for ecosystem service

We found that forests and shrubs were effective in preventing soil erosion, enhancing sandstorm prevention, with an average soil conservation capacity of forests of 373.81 t km^−2^, and average sandstorm prevention capacity of shrubs of 1,703.06 t km^−2^ (Table 2) Moreover, grassland played a vital role in sandstorm prevention, with an average sandstorm prevention capacity reaching 3,392.99 t km^−2^. Wetlands had a high value for water conservation, with an average water yield of 225.26 × 10^3^ m^3^ km^−2^.

**Table 2:** Ecosystem service function of various land types (per unit area).

	Soil conservation (t/km^2^)	Water yield 10^3^ m^3^/km^2^)	Sandstorm prevention (t/km^2^)	Diversity (species/km^2^)
Forest	373.81	60.63	1,415.87	30.22
Shrub	302.55	58.26	1,703.06	30.50
Grassland	130.39	50.65	3,392.99	26.96
Wetland	0.00	225.26	0.00	6.18
Farmland	30.76	36.53	1,244.03	2.42

### Spatial distribution of ecosystem services in the BHT Region

**Figure 4:**
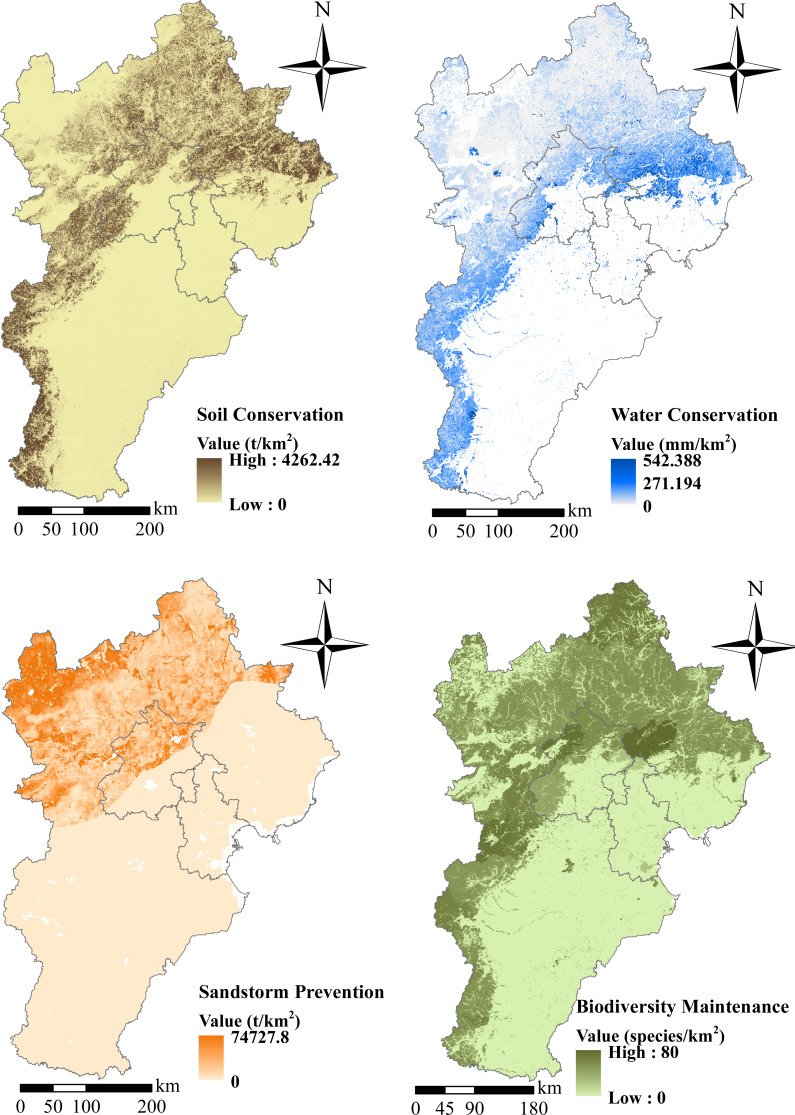
Spatial distribution of ecosystem services in the Beijing-Tianjin-Hebei region. (A) Soil conservation. (B) Water conservation. (C) Sandstorm prevention. (D) Biodiversity maintenance.

The Yanshan Mountains in the north of the BTH region and the southwestern part of the Taihang Mountains have the strongest soil conservation (Fig. 4). Areas in the northeast and southeast of the region were identified for potential service of water and soil conservation, and were typically farmlands and grasslands. The distribution of water conservation service areas overlapped with those of soil conservation, but the most important areas for water conservation service were found on the fringes of cities, including Beijing. ES of sandstorm prevention measures were important in the northwestern part of the BTH region, with the rest of the area less important. In addition, half of Beijing was included as areas of sandstorm prevention services. The highest species richness was found in areas surrounding Beijing, Tianjin, and Baoding, with some areas of high species richness in the plains.

The spatial distributions of ES and biodiversity were notably different (Fig. 4). The ecosystems in the northern part of the region, which are typically forest and grassland mountainous areas, provide more valuable services than those in the eastern agricultural plains, such as soil conservation, water yield (values >30%), and sandstorm prevention. The species richness is high in the northern mountainous areas, which also had fewer anthropogenic disturbances.

According to our data, the ES in the BTH region have improved significantly (Table 3), with soil conservation reaching 3.0 × 10^7^ t and water yield reaching 4.24 × 10^9^ m^3^. In addition, sandstorm prevention capacity increased with the quantity of lost sand decreasing 2.92 × 108 t. Moreover, opportunities for promoting ES in the BTH region are numerous. Forests were best at soil conservation, while shrubs and grasslands were next best. Grasslands played the most important role in sandstorm prevention, while shrublands, forests, and farmland were less important.

**Table 3:** Features of ES function in Beijing-Tianjin-Hebei region.

Types	Total soil conservation (10^6^t)	Total water flood (10^6^m^3^)	Total windbreak and sand fixation (10^6^t)	Total biodiversity (score)
Forest	17	2,697	63	1,342,920
Shrub	7	1,441	42	753,481
Grassland	3	993	66	528,755
Wetland	0	11	0	34,485
farmland	3	8	121	237,153

### Hotspots of ecosystem services in the BTH Region

**Figure 5:**
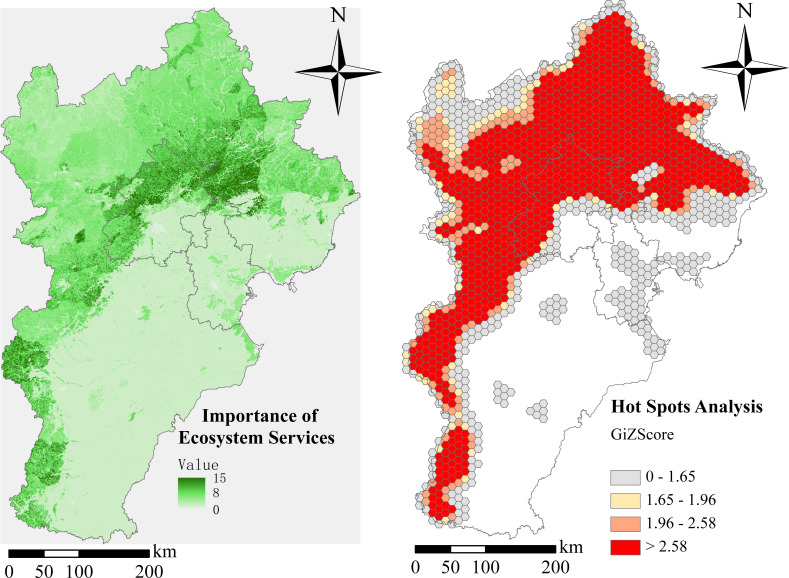
Ecosystem services hotspot analysis in Beijing-Tianjin-Hebei region. (A) Importance of ecosystem services. (B) Hot spots analysis.

Our team combined and studied several ES (soil conservation, water conservation, sandstorm prevention, and biodiversity importance) by the method described above. Our results show that the higher the regional vegetation coverage rate is, the stronger the function of ES in hotspot areas is. Areas of important distribution stretched through the Taihang and Yanshan Mountains (Fig. 5A). The Yanshan Mountains play the most important role in the northern BTH region and their proximity to Beijing is vital for maintaining the local ecosystem within the capital region. There are also very important ES distribution areas in the western and southwestern BTH region. Farmland, found mainly in the southern and eastern regions, was of little ecological importance.

A high concentration of ES hotspots was found mainly in the northern and southwestern parts of the BTH region, due primarily to the presence of national nature reserves with high biodiversity (Fig. 5B). Compared with other regions, variability increased the degree of spatial aggregation and the absolute value of G_i_*.

## Discussion

### Relationships between biodiversity and ecosystem services

The soil retention service function was mainly distributed in the Yanshan Mountains in the northern part of the BTH region and the Taihang Mountains in the southwestern part of the region. The distribution of the water conservation function was basically the same as that of the soil retention function. Areas with high importance of ES were mainly distributed in the northwestern part of China (Gou et al., 2020). These areas have limited access and are not suitable for agricultural development; however, they are especially important for the ecosystem structure of China. The value of ES was lower in the central and southeastern plains areas. The main reason may be that this area concentrates agricultural development in China, and large tracts of cultivated farmland cause shortages of groundwater resources in the area, slowing the recovery rate of natural vegetation. Further, the area is densely populated, and it has convenient transport, low altitudes, and strong human disturbance. The importance of ES is relatively low in the central plains and southeastern BTH region, because this area is dominated by plains, and the main ecosystem types of the area are farmland and grassland (Gou et al., 2021).

The spatial distributions of ES and biodiversity were notably different. Overall, the northern mountain forest and grassland ecosystems typically provide more valuable soil and water conservation, water retention, and sandstorm prevention services than the eastern agricultural plains. Species are more abundant in the northern high mountains with less human interference than elsewhere. Important distribution areas of the northern mountain ecosystems were mainly in the northwestern part of China, with grassland as the main ecosystem type in the area. The possible reason for that is the dominant wind direction in the area is northwesterly and mainly prevalent in autumn and winter; at those time, vegetation plays an important role as sandstorm prevention even though vegetation cover is low.

The northern and western mountains of the BHT region often have extremely rich species diversity due to their unique and complicated environmental and climatic conditions. Habitats for species to spread and distribute often ranges in large mountain. However, due to increasing human activities, habitats are gradually shrinking, which is insufficient to protect species populations and natural ecological processes in the long run. Therefore, there is an urgent need to integrate habitat into a larger spatial scale and to protect, restore and strengthen ecological connectivity between habitats. The construction and restoration of habitats are considered to be the key to the fundamental realization of ES management. The spatial distribution of existing habitat protection systems is not well matched with the spatial distribution of biodiversity. So there is a huge gap in the adequate representation of biodiversity in habitat protection. To address this shortcoming, we propose the creation of a new type of protected area to improve biodiversity and habitat quality. In order to expand the political viability of habitats, it is essential to allow some allowable use of natural resources as long as it does not compromise biodiversity and habitat quality goals.

### The importance of the selection of important ES areas

ES hotspot maps can be used as a visual and vivid tool to initiate communication with stakeholders on management planning. Identifying hotspots can help prioritize the maintenance of basic ES in limited financial resources. ES hotspots should be reserved first to avoid being destroyed. In this paper, hotspots mainly appear in the western and northern regions of BHT with high vegetation coverage. While our ecosystems are often complex and a landscape often has multiple services, multiple ESs should be considered at once to preserve multifunctional hotspots. Gi* statistical-based hotspot analysis works well for this situation as well. Ecological restoration is the process by which humans help restore a degraded ecosystem, with the goal of restoring it to its original state or a new state. In the BHT region, ecological restoration has become especially important to limit the human influence and protect the geographical space of nature. Now that ecological restoration is undergoing a major shift, expanding the goal from focusing primarily on biodiversity to also including the provision of ES for human wellbeing, how to achieve both biodiversity and ES is a top priority.

Additional areas with high importance of ES were the Taihang and Yanshan Mountains. The Yanshan Mountains in the northern BTH region play an important role in the maintenance of ecological security of the Beijing area. The southern and eastern parts of the region are mainly farmland with little ecological importance (Wu et al., 2017). The strengthening of biodiversity conservation and ecosystem restoration in the region may effectively improve habitat quality and ES, and support the development of agriculture and economy in the northeastern part of China. This work will expand areas of biodiversity conservation and set priorities for further expansion of existing nature reserves for threatened species protection and to provide ES. For example, the conservation areas near Beijing and the Taihang Mountains are to be expanded.

Multiservice areas of biodiversity change were concentrated in the northern and southwestern parts of the BTH region. Due to the existence of national nature reserves in the vicinity of multiservice areas, biodiversity was high, and the changes were more intense than in other areas, resulting in a higher degree of spatial aggregation and higher absolute value of G_i_*. Given the misalignment between biodiversity and regulatory services, expanding existing nature reserves and establishing new ones is an important step. In short, the expansion of existing nature reserves and the establishment of new nature reserves in these areas will also protect more areas of important ES. Based on experiences in China and in other regions, biodiversity and ES are useful measures for determining important areas for the expansion of ecological reserves, which will improve local and national support for conservation investment, effectiveness, and sustainability (Miao et al., 2019).

ES mapping can provide guidance for conservation policy formulation, but we suggest that hotspots analysis should be integrated into priority regional settings for system protection. Priority sites with ES hotspots are considered comprehensive, compact, and cost-effective. In practice, conservation budgets are often insufficient to protect all sites. To be cost-effective, hotspots must be compact and have a low edge-to-area ratio. But the hotspot analysis method based on Gi* statistics is one of the quantitative methods of spatial clustering, which is particularly effective for evaluating and identifying ES hotspots with good spatial connectivity. Therefore, this approach is more conducive to practical and cost-effective ES conservation management.

### Research methods and policy implications

We only analysis four ES which do not adequately reflect the ecosystem pattern of the area. Moreover, identifying an area of ecosystem research in which there are significant uncertainties (Li et al., 2022). Several empirical parameters, such as the rainfall erosion rate, soil conservation measure and annual precipitation, increase the uncertainty of the results. According to the previous studies, we determine the values of these parameters (Li et al., 2021). Therefore, it is necessary to analysis more ES and compare with each other. More research should be done to improve the assessment methods.

Gi* statistics is one of the spatial clustering methods that works by computing the local sum of a feature and its neighbors, and then comparing the initial result proportionally to the sum of all features. A statistically significant *Z*-score (*i.e.*, Gi) is output when the computed local sum is completely different than expected, and the difference exceeds random chance, so Gi * Statistic is a more robust method for identifying hotspots. Additionally, geostatistical analyses such as Moran’s I, Getis-Ord Gi* statistics can also be used to identify hotspots, but in practice, Getis-Ord Gi* statistics (or simply Gi* statistics) have proven to be a superior alternative.

Identification of ES hotspots can define conservation targets and help establish priority sites for ecosystem management and allocation of limited resources. We propose two methods for identification of hotspots. One method involves defining certain thresholds. For instance, Gimona stated that when a grid value of ES is higher or lower than a median grid value, hotspots can be differentiated. However, this method has a disadvantage: it has low efficiency for identification of ES hotspots with spatial connectivity. The other method focuses on spatial cluster analysis, in which kernel density estimation (KDE) may show the location of centralized points or line features. In addition, Getis–Ord G_i_* statistical data could be used to identify hotspots, because this method considers all adjacent features and the value of hotspots of different statistical significance. The hotspot output can display ranking of sufficient landscape connectivity in succession (Sun et al., 2019).

Reinforcement of biodiversity conservation and ecosystem restoration in ES hotspots in the BTH region may effectively improve habitat quality and ES, support agriculture and economic development in the southeastern part of the region, and enable the coexistence of development and environmental protection (Zhang et al., 2017). Areas that are important for conservation of biodiversity and for provision of different ES are not always well matched, and, in fact, many are misaligned (Fu & Wei, 2018). Nature reserves will not expand in these areas, therefore, deficiencies in the protection of ES will also remain unaddressed. To this end, we should build a large number of protected areas in our BHT hotspot areas or importance ES areas or delineate ecological red lines in these areas. Large-scale human activities are prohibited in these areas. Only in this way can the biodiversity and ES be enhanced. Both the expansion of biodiversity preservation areas and allowances for the use of natural resources are essential, as long as the ES objective is not compromised. ES and biodiversity contribute to the balance between economic development and conservation of natural resources in the BTH region (Xie, He & Xie, 2017).

## Conclusions

This study provides a spatial framework for addressing priorities in ES protection. Stakeholders can integrate this approach into their own frameworks to identify and preserve multiple functional hotspots of ES or biodiversity, thus supporting targeted ES policy making. Our results show that the key ES areas are mainly distributed in the Yanshan and Taihang Mountains in the southwestern BTH region. Our work discusses the spatial features of ES in related areas. The spatial distribution of soil and water conservation areas is in line with that of biodiversity, and the mountainous areas northwest of Beijing play a critical role in that biodiversity. The main ecosystem types in Tianjin and Hebei are forests and shrubs.

The lower slopes of the Yanshan Mountains in the BTH region are important to protect the ecosystem of the capital region. Hotspots rich in biodiversity are concentrated in the northern and southwestern parts of the BTH region, which are close to national nature reserves. Biodiversity changes are more intense and concentrated in those areas than in other regions. This study establishes the importance of multiservice ES areas in the BTH region, and shows that intensification of biodiversity conservation and ecosystem restoration in the region can effectively improve habitat quality and ES, support agricultural and economic development in the southeastern part of the region, and help in achieving the coexistence of development and environmental protection.

##  Supplemental Information

10.7717/peerj.13881/supp-1 Supplemental Information 1 Land use of Beijing-Tianjin-Hebei in year 2015 Beijing-Tianjin-Hebei Region and distribution of land use in year 2015 (Arcgis Desktop software).

10.7717/peerj.13881/supp-2 Supplemental Information 2 Land cover data of Beijing-Tianjin-Hebei Region for 2000 and 2010 Arcgis software.

10.7717/peerj.13881/supp-3 Supplemental Information 3 Assessment of ecosystem service

10.7717/peerj.13881/supp-4 Supplemental Information 4 Ecosystem services hotspot analysis in Beijing-Tianjin-Hebei Region

## Additional Information and Declarations

### Data Availability

The following information was supplied regarding data availability: Raw data are available in the Supplementary Files.
