# Supplementary figures and images for: Identifying important ecosystem service areas based on distributions of ecosystem services in the Beijing–Tianjin–Hebei region, China
Source: PeerJ. 2022 Aug 18;10:e13881. doi: 10.7717/peerj.13881 (PMC9393009; doi:10.7717/peerj.13881)

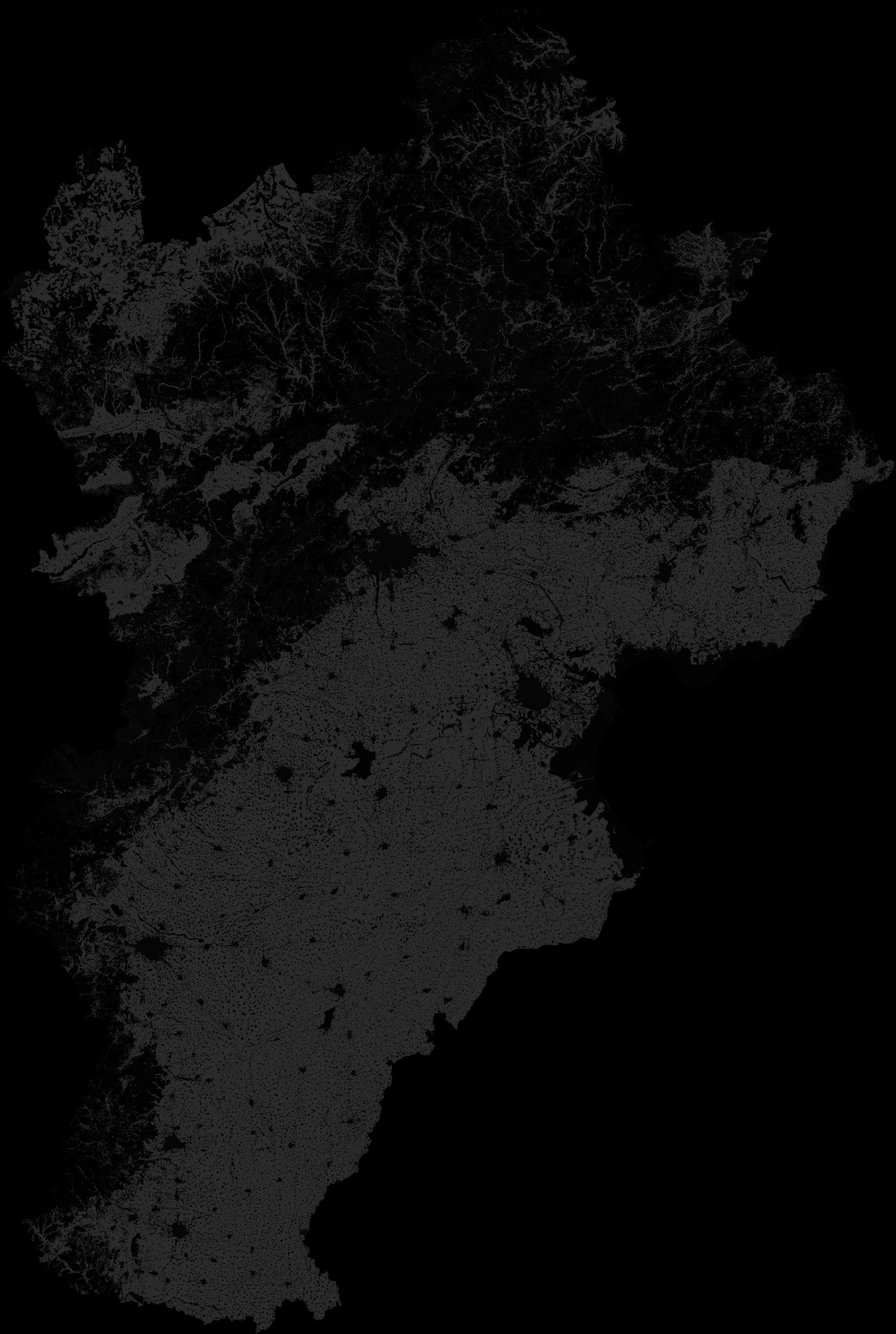

Supplement: Supplemental Information 2 — Arcgis software. [file peerj-10-13881-s002.zip › Land-use and land-cover of Beijing-Tianjin-Hebei in 2000 and 2010/2000.tif]

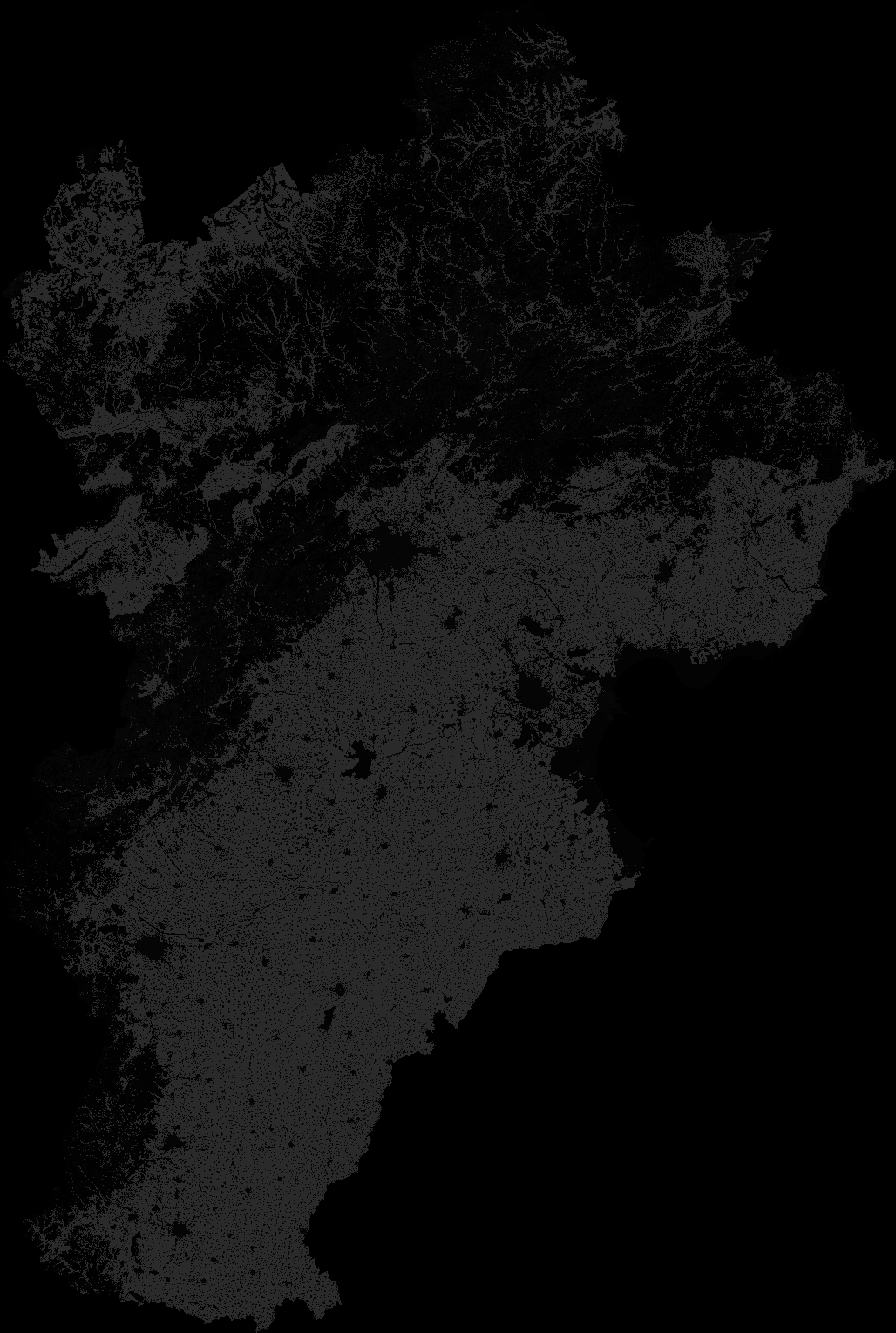

Supplement: Supplemental Information 2 — Arcgis software. [file peerj-10-13881-s002.zip › Land-use and land-cover of Beijing-Tianjin-Hebei in 2000 and 2010/2000.tif.ovr]

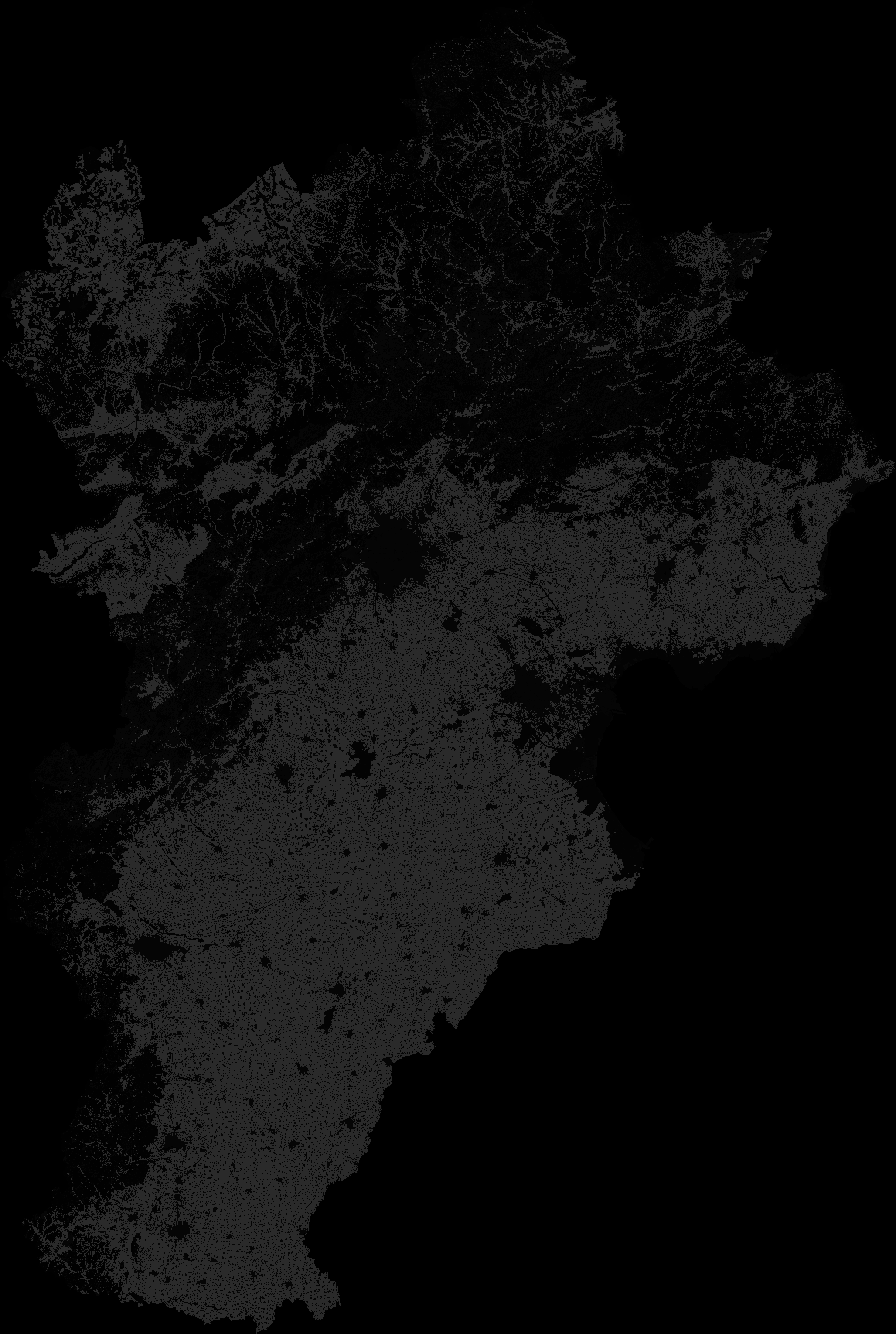

Supplement: Supplemental Information 2 — Arcgis software. [file peerj-10-13881-s002.zip › Land-use and land-cover of Beijing-Tianjin-Hebei in 2000 and 2010/2010.tif]

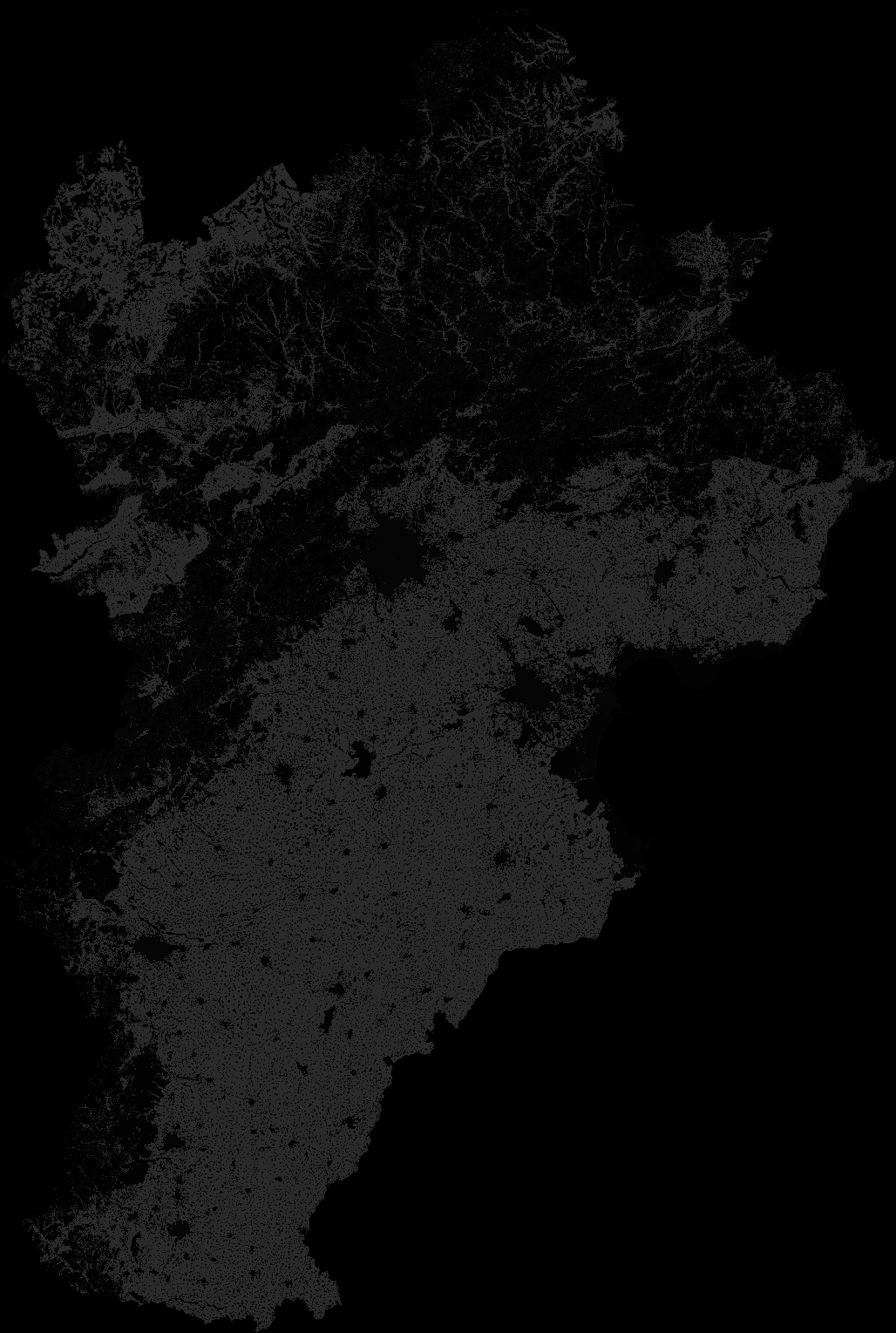

Supplement: Supplemental Information 2 — Arcgis software. [file peerj-10-13881-s002.zip › Land-use and land-cover of Beijing-Tianjin-Hebei in 2000 and 2010/2010.tif.ovr]
